# A germline c.1546dupC MEN1 mutation in an MEN1 family: A case report

**DOI:** 10.1097/MD.0000000000026382

**Published:** 2021-06-25

**Authors:** Yoon Young Cho, Yun Jae Chung

**Affiliations:** a Division of Endocrinology and Metabolism, Department of Medicine, Soonchunhyang University Bucheon Hospital, Bucheon; b Division of Endocrinology and Metabolism, Department of Internal Medicine, Chung-Ang University, College of Medicine, Seoul, Korea.

**Keywords:** c.1546dupC, germline mutation, Korea, multiple endocrine neoplasia type 1, phenotype

## Abstract

**Rationale:**

Multiple endocrine neoplasia type 1 (MEN1) is a rare tumor syndrome with an autosomal dominant inheritance, and genetic testing for *MEN1* gene is important for both affected individuals and their relatives. We present a 2-person family affected by a germline c.1546dupC *MEN1* mutation, and one of them had a full-spectrum of MEN-related endocrine tumors.

**Patient concerns:**

A female patient aged 32 years presented with jejunal ulcer perforation due to gastrinoma.

**Diagnoses:**

We conducted genetic analysis and extensive biochemical/radiological evaluation for detecting other endocrine tumors. Multiple pancreatic neuroendocrine tumors (NETs), prolactinoma and primary hyperparathyroidism were diagnosed, and a frame-shift mutation, NM_130799.1:c.1546dupC (p.Arg516Profs∗15), was detected. One daughter of the proband, aged 12 years, had the same mutation for MEN1.

**Intervention:**

She underwent pancreatic surgery for pancreatic NETs and total parathyroidectomy for primary hyperparathyroidism.

**Outcomes:**

After pancreatic surgery, long-term symptoms of epigastric soreness, acid belching, sweating, and palpitation in fasting were improved. Hypercalcemia was improved after parathyroidectomy and she was supplemented with oral calcium and vitamin D. Her daughter showed normal biochemical surveillance until 15 years of age.

**Lessons:**

We report 2 people in a family affected by MEN1 with the heterozygous germline c.1546dupC mutation, a variant that should be surveilled for early development of full-blown MEN1-associated endocrine tumors.

## 1 Introduction

Multiple endocrine neoplasia (MEN) type 1 (MEN1) is a rare tumor syndrome with an autosomal dominant inheritance and high penetrance >95% by age 40 to 50 years.^[1]^ Diagnosis of MEN1 is based on clinical features of at least 2 of 3 following endocrine tumors: parathyroid adenoma, anterior pituitary tumor, and pancreatic neuroendocrine tumor (NET). Penetrance of main MEN1 tumors by age 50 years is highest for the parathyroid glands (>95%), followed by the pancreas (30%–70%) and the anterior pituitary gland (30%–40%).^[2]^

In 1997, a clone of the gene causative of MEN1 that consists of 10 exons encoding a 610-amino acid protein referred to as menin was identified (GenBank Accession No.: U93236.1). As a tumor suppressor gene, the *MEN1* gene functions mainly as loss of heterozygosity at the menin locus of chromosome 11q13,^[3,4]^ and present genetic testing for the *MEN1* gene focuses on detection of the polymorphic markers in this region. More than 1300 *MEN1* mutations have been identified,^[1,5]^ and >70% of these mutations are related to truncated forms of menin: frame-shift mutations (42%), nonsense mutations (14%), exon region deletion due to splicing defects (10.5%), or large deletions (2.5%) lead to pathologic consequences.^[5,6]^ In contrast, missense mutations (25.5%) and single or few amino acid in-frame deletions or insertions (5.5%) do not predict obvious inactivation of menin; instead, these mutations potentially affect proteins interacting with menin or promote the rapid degradation of menin.^[5,6]^ Moreover, classification of these mutations as benign or pathologic needs further verification in affected families.

Genetic diagnosis of MEN1 has clinical importance for reducing disease-related morbidity and mortality of index cases and their relatives. In particular, early biochemical surveillance for genetically affected asymptomatic relatives can help to detect MEN1-related tumors even 10 years before clinically evident disease.^[6]^ In contrast, the negative result of genetic testing for *MEN1* mutations can eliminate the psychological burden of possible MEN1 in patient relatives. Thus, genetic testing is recommended in first-degree relatives of index patients.^[2]^

In this report, we present 2 people in a family affected by MEN1 caused by a frameshift mutation, NM_130799.1:c.1546dupC (p.Arg516Profs∗15). The c.1546dupC mutation has been reported as a pathologic *MEN1* mutation in genetic studies for MEN1^[7–10]^; however, clinical manifestations of affected individuals had not been reported in detail. We report a 32-year-old female who had 3 common MEN1 tumors; in particular, she had various kinds of pancreatic NETs, 3 insulinomas, 1 glucagonoma, and 1 gastrinoma, simultaneously. One 12-year-old daughter of the index patient had the same *MEN1* mutation but has shown no clinical evidence of MEN1.

## 2 Materials and methods

Laboratory tests, imaging studies, and pathological assessment were performed at the Chung-Ang University Hospital. Genetic testing was conducted on June 2015 by the Green Cross Genome (Yongin 16924, Korea). DNA was extracted from the peripheral blood samples of the index patient and her daughter. Sanger sequencing of the MEN1 gene demonstrated duplication of the 1546th base in exon 10 (NM_130799.1:c.1546dup), resulting in frame-shift mutation (p.Arg516Profs∗15). The result was compared with several databases (1000GP, ClinVar, Exome Variant Server, FATHMM, Gene Ontology Resource, Genome-wide Association Study, HGMD, PolyPhen-2, and PROVEAN). The patient and her daughter provided their written consent for anonymous use of their medical data for scientific publication.

### 2.1 Case report

A 32-year-old female was referred to our emergency department for severe abdominal pain due to panperitonitis. She had undergone laparoscopic simple closure for jejunal ulcer perforation. She had experienced one similar episode of panperitonitis and emergent surgery at an outside hospital 1 year before. Two years after the birth of her daughter 12 years before, the patient had experienced amenorrhea and galactorrhea.

She also had suffered from epigastric soreness and acid belching for 5 years and sweating and palpitation in fasting for three years. Symptomatic hypoglycemia with elevated serum insulin (63.58 mU/L; reference ranges, 3–25 mU/L) and c-peptide (4.34 ng/mL; reference ranges, 0.81–3.85 ng/mL) was detected at admission. In addition, serum chromogranin-A (260 ng/mL; reference ranges, 0–108 ng/mL) and gastrin (536 pg/mL; reference ranges, 25–111 pg/mL) levels also increased.

**Figure 1:**
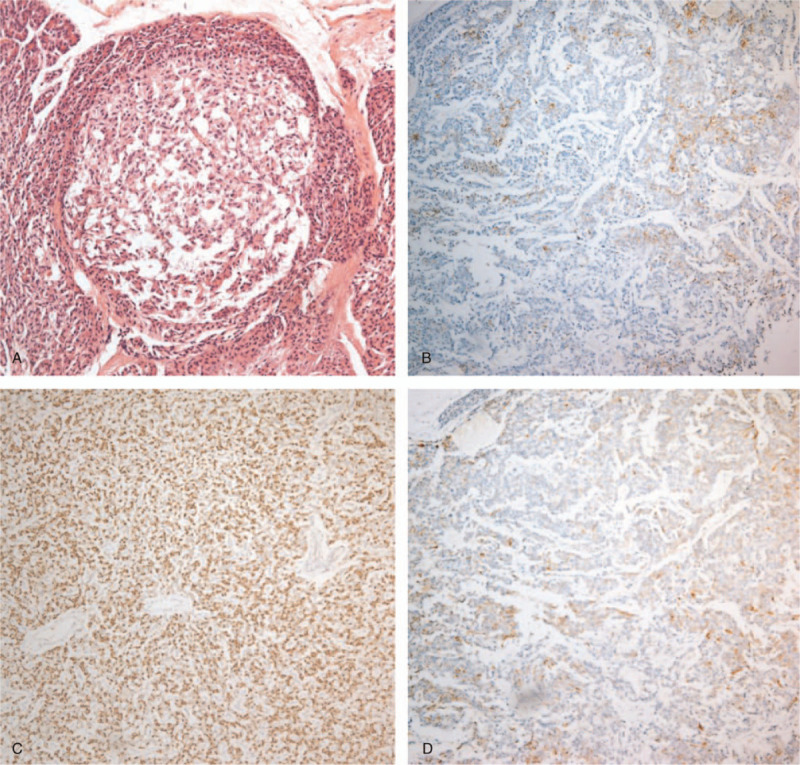
Low-power view of pancreatic neuroendocrine tumor (A) and immunostaining for each hormone (B–D). (A) Well-differentiated neuroendocrine tumor that characterized by small round to oval nuclei with “salt and pepper” chromatin and eosinophilic cytoplasm. (B) Positive staining for insulin in a pancreatic tail tumor. (C) Positive staining for glucagon in a pancreatic body tumor. (D) Positive staining for gastrin in a pancreatic tail tumor.

With the strong suspicion of MEN1, extensive work-up for detecting endocrine tumors and the genetic testing was conducted. After excision of pancreatic masses, the patient was confirmed to have 5 pancreatic NETs with hypersecretion of insulin and gastrin by the staining for immunohistochemistry (Fig. 1). Fasting hypoglycemic symptoms and epigastric soreness were much improved after surgery. She had been taking a proton pump inhibitor and was regularly assessed using pancreas using pancreas computed tomography (CT). However, 1 year later, a new pancreatic head mass appeared, and serum gastrin (195 pg/mL; reference ranges, 25–111 pg/mL) and chromogranin-A (865 ng/mL; reference ranges, 0–108 ng/mL) concentrations increased again.

**Figure 2:**
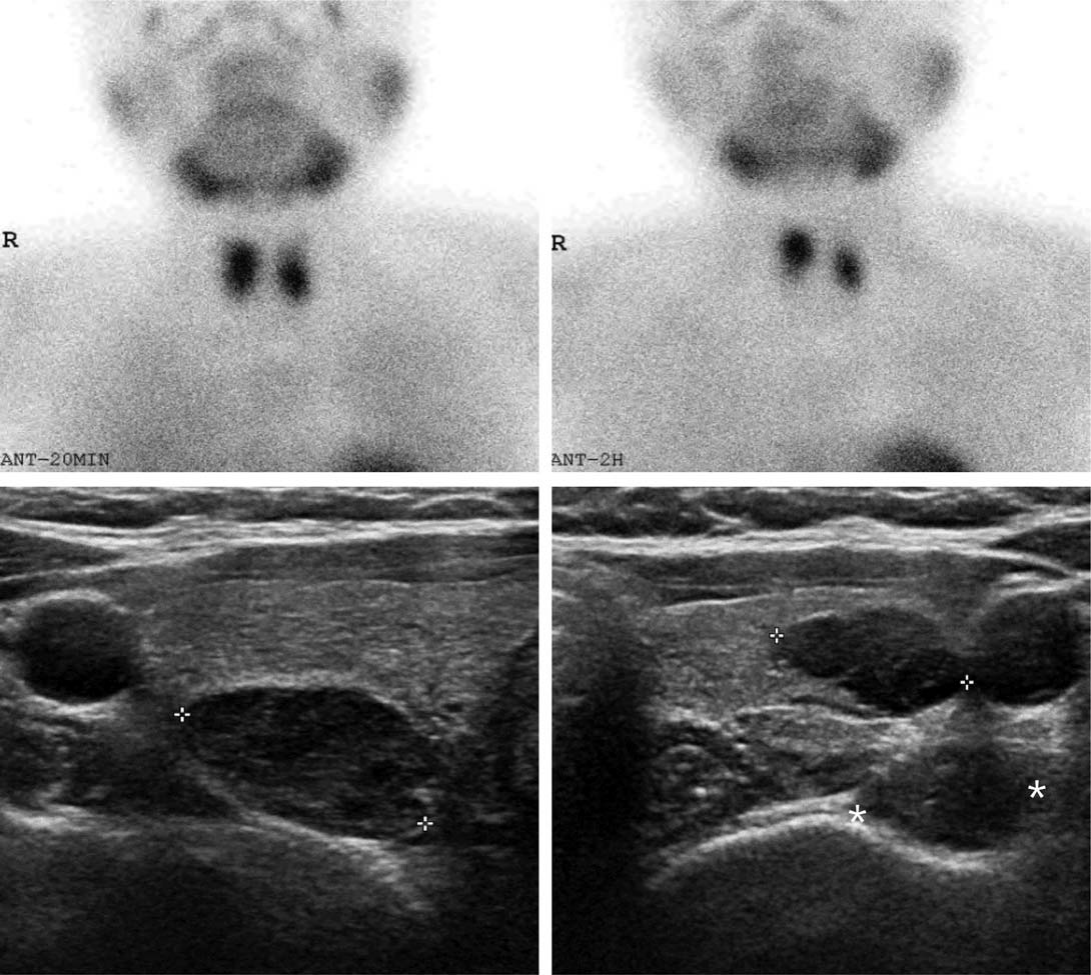
Right and left inferior parathyroid adenomas detected on ^99^Tc-sestamibi parathyroid scintigraphy (upper) and thyroid ultrasonography (lower).

Primary hyperparathyroidism was diagnosed based on serum hypercalcemia (serum calcium, 12.6 mg/dL; reference ranges, 8.8–10.6 mg/dL) and elevated parathyroid hormone (PTH) concentration (serum PTH, 459 pg/mL; reference ranges, 14–72 pg/mL). Symptoms of hypercalcemia were not evident; however, severe osteoporosis with a *z*-score of −3.0 in the lumbar spine and −2.1 in the femur was diagnosed based on bone mineral density (BMD). Two parathyroid adenomas were detected on thyroid ultrasonography and ^99^Tc-sestamibi parathyroid scintigraphy, preoperatively (Fig. 2). A few months after the pancreatic surgery, she underwent total parathyroidectomy with auto-transplantation on the left forearm and was supplemented with oral calcium and vitamin D.

**Figure 3:**
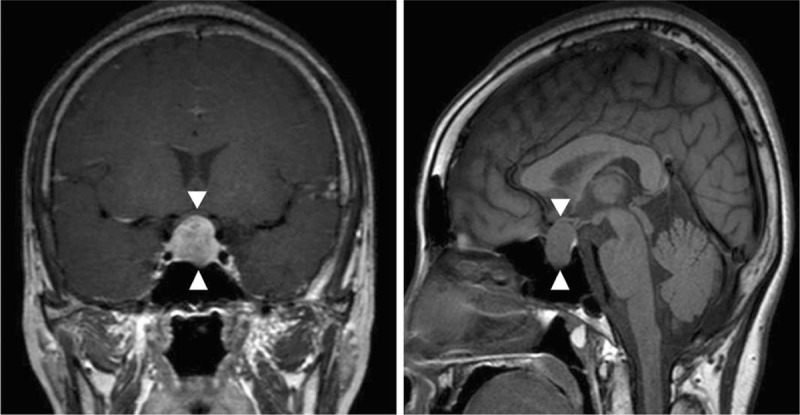
Pituitary macroadenoma with suprasellar extension detected on contrast-enhanced magnetic resonance imaging.

Hyperprolactinemia (serum prolactin, 2067 ng/mL; reference ranges, 3–35 ng/mL) and a 1.6 × 1.3 × 1.6-cm-sized pituitary adenoma with suprasellar extension had led to diagnosis of prolactinoma (Fig. 3). She had been medically treated with cabergoline.

Genetic testing revealed the heterozygous germline mutation c.1546dupC in the index patient and her 12-year-old daughter. The daughter had no clinical evidence of MEN1 until 15 years of age. In addition, the patient reported that her father had died of pancreatic cancer at 65 years of age.

## 3 Discussion

In this report, we present a young female patient with a full spectrum of MEN1-associated tumors and the heterozygous c.1546dupC germline mutation. At genetic diagnosis of MEN1, she harbored three of the most common MEN1 tumors: parathyroid adenomas, pancreatic NETs, and prolactinoma. In spite of sustained symptoms of pancreatic NETs and prolactinoma for nearly a decade, she was transferred to our hospital for jejunal ulcer perforation and was confirmed to have multiple and symptomatic NETs comprised of insulinomas, glucagonoma, and gastrinoma. Among pancreatic NETs, gastrinoma developed most frequently (40%) in MEN1, followed by insulinoma (10%), glucagonoma (<1%), and Vasoactive intestinal peptide tumors (VIPomas, <1%).^[2]^ Our index patient experienced symptoms of prolactinoma in her early 20s, along with symptoms for gastrinoma and insulinomas. Considering that approximately 50% of affected individuals experience MEN1-related symptoms by 20 years of age, 95% by 40 years, and 100% by 60 years,^[11,12]^ she showed a full spectrum of MEN1-related tumors early in life. Her affected 12-year-old daughter had no clinical evidence of MEN1 tumors on regular follow-up.

In the literatures, no distinct genotype-phenotype correlation has been reported for MEN1. Carriers of the same *MEN1* gene show different tumor penetration and malignant transformation.^[13]^ Marked differences in MEN1 phenotypes have been reported even in identical twins.^[14–16]^ In the present report, the index patient developed MEN1-related symptoms early in her life. For this reason, regular biochemical and radiological screening should be conducted for the affected daughter to determine if she has a similar phenotype of MEN1 to her mother. One previous case report of the sporadic MEN1 case mutated with c.1546dupC in a 20-year-old female presenting with Cushing disease and primary hyperparathyroidism^[17]^ showed the possibility of early development of MEN1-related tumors in the affected individuals with the c.1546dupC mutation.

The germline mutation causative for MEN1 should be documented in the first-degree relatives to determine pathogenicity. The father of the present patient had died for pancreatic cancer before the index patient was confirmed as an MEN1 patient. As the malignant pancreatic NET is an important cause of death for MEN1 patients,^[18]^ the farther might have been a gene carrier; however, genetic information was not available. The daughter of the patient had the same *MEN1* mutation, confirming that the c.1546dupC mutation is a germline mutation with an autosomal dominant inheritance.

Among >1300 mutations of MEN1, the sites of the 9 germline mutations show frequency >1.5% and collectively represent approximately 20% of all reported germline mutations.^[1]^ Among the 9 frequently reported mutations, 2 frame-shift mutations (c.1546delC and c.1546_1547insC) were located on exon 10, codon 516,^[1]^ and the c.1546_1547insC mutation also is known as c.1546dupC. However, previous studies regarding *MEN1* mutations had reported the c.1546dupC mutation as one of dozens of detected mutations^[7–10]^; thus, only 1 case of detailed phenotypes of the c.1546dupC mutation has been described.^[17]^ Our report has the meaning in description of the diversity and severity of clinical manifestations of MEN1 caused by the c.1546dupC mutation.

Genetic testing for MEN1 is important due to a 50% risk of inheritance in the first-degree relatives of the patients with MEN1. Untreated patients with MEN1 are expected to have the decreased life expectancy, with a 50% probability of death by the age of 50 years.^[18,19]^ The major causes of death are malignant tumors (ie, malignant pancreatic NETs) or sequelae of the diseases,^[18,20]^ although, the prognosis of MEN1 patients had been improved after the use of acid-suppressive agents for gastrinoma.^[18]^ Therefore, early detection of the preclinical diseases can improve the prognosis of affected individuals through appropriate treatment specific for MEN1 tumors. In addition, negative genetic results in the family members of affected individuals relieve the burden of lifelong surveillance for the diseases.^[2]^

In conclusion, we report a young female patient and her daughter with a frame-shift germline c.1546dupC mutation, and the phenotype has the possibility of the early development of full-blown MEN1-associated endocrine tumors.

## Author contributions

**Conceptualization:** Yun Jae Chung

**Data curation:** Yoon Young Cho and Yun Jae Chung

**Formal analysis:** Yoon Young Cho and Yun Jae Chung

**Investigation:** Yoon Young Cho

**Methodology:** Yoon Young Cho

**Resources:** Yun Jae Chung

**Supervision:** Yun Jae Chung

**Writing – original draft:** Yoon Young Cho

**Writing – review & editing:** Yoon Young Cho and Yun Jae Chung
